# A 14-year literature survey on spine-related clinical research output by orthopedic surgeons from mainland China

**DOI:** 10.1097/MD.0000000000011959

**Published:** 2018-08-24

**Authors:** Gao Si, Xiao Liu, Nanfang Xu, Miao Yu, Xiaoguang Liu

**Affiliations:** a Orthopaedic Department, Peking University Third Hospital; b Peking University Health Science Center, Haidian District, Beijing, China.

**Keywords:** bibliographical study, mainland china, medical subject heading terms, spine surgery

## Abstract

**Background:**

In recent years, China is increasingly playing an active role in various fields of biomedical research. Many bibliometric studies have provided valuable insights to different fields of clinical studies. However, similar evaluation on spine surgery-related clinical research is still limited. We herein aimed to examine the scientific publications by orthopedic spine surgeons from mainland China within a 14-year period.

**Methods:**

Articles were identified in PubMed using predetermined query terms. Descriptive statistics were calculated, and *T* tests, Chi-squared tests, and regression analysis were conducted on the number of publications, impact factors (IFs), citations, region of the study, and associated medical subject headings (MeSHs).

**Results:**

A total of 1498 articles were identified and the annual number of publications, citations, and IFs all increased exponentially. The average IF was significantly higher in 2007 to 2013 than 2000 to 2006. Most publications were from Shanghai and Beijing and the 5 most productive administrative regions generated 70% of all publications. Analysis of associated MeSHs suggested research topics became more heterogeneous over the study period.

**Conclusion:**

This was the first comprehensive evaluation on the clinical research output by orthopedic spine surgeons from mainland China. The annual number of publications and citations both increased significantly; however, research was highly concentrated in a handful of administrative regions.

## 1 Introduction

Publication of scientific study results is an efficient way to disseminate knowledge and improve communication between researchers,^[1]^ and it represents an important indicator for research contribution. Bibliometric research on scientific publications not only can be used to quantitatively evaluate the contribution of individual researchers, academic group, research institute, and research productivity of a country in a specific field of study,^[2,3]^ but also can provide supporting evidence for policy and decision making.^[4]^ Now bibliometric analyses have been published in a wide range of scientific fields, such as cancer,^[5]^ diabetes,^[6]^ cardiovascular disease,^[7]^ respiratory medicine,^[8]^ tuberculosis,^[2]^ stem cells,^[9]^ exosome,^[10]^ public health,^[11,12]^ etc. There have been some bibliometric analysis of global spine research or orthopedic research.^[3,13–16]^ However, bibliometric evaluation on the contribution to clinical research related to orthopedic spine surgery from mainland China was still limited.^[17,18]^ In this study, we aimed to examine the available literatures in orthopedic spine surgery from mainland China in English publications within a 14-year period.

## 2 Methods

### 2.1 Search strategy and data extraction

**Figure 1:**
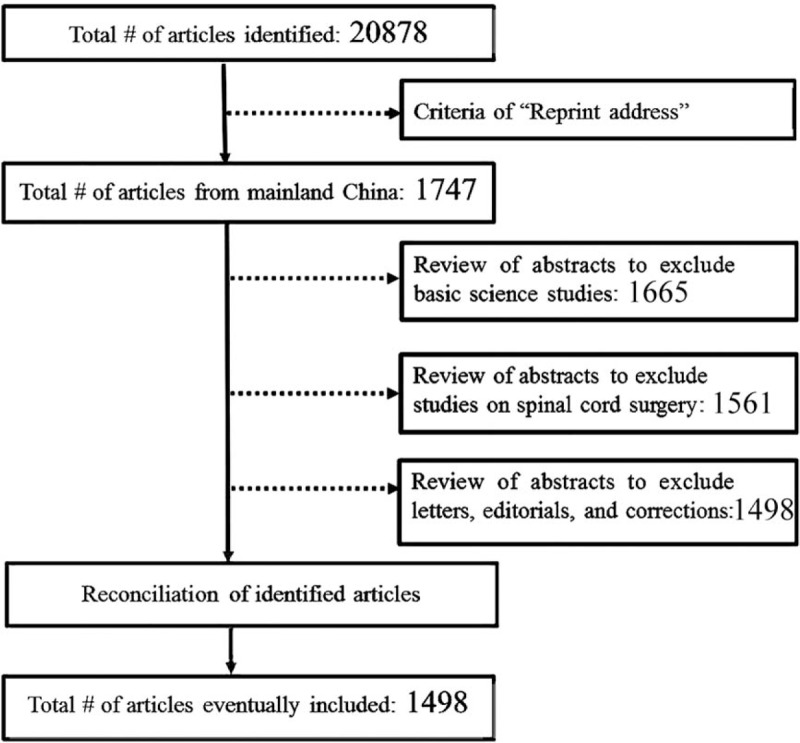
Flowchart on the identification of relevant articles included in the review.

A computerized literature search was performed in the online database PubMed (http://www.ncbi.nlm.nih.gov/pubmed/) in March 2015 following the PRISMA guidelines^[19,20]^ to identify the relevant literature published between 2000 and 2013. The query term “(spine[Title/Abstract] OR spinal[Title/Abstract]) AND surgery[Title/Abstract]” was used in combination with the filter settings of “Species: Human” and “Language: English.” The “Reprint address”^[1]^ for each article was manually checked to be consistent with institutes within mainland China. The abstracts of the resulting articles were further reviewed to exclude all basic science studies. Letters, editorial material, and correction were excluded. Articles were selected independently by 2 reviewers and disagreement was resolved by discussion and consultation with a third reviewer when necessary (Fig. 1). Year and journal of publication as well as the province where the research was performed were noted when available from the PubMed database^[7]^ for each article. The impact factor (IF) of each journal and relevant citation information was extracted from the yearly updated versions of journal citation reports (JCRs) by Thomson Reuters through the Peking University Health Science Library's Journal Citation Reports (online). All analyses were based on previous published studies, thus no ethical approval and patient consent are required.

### 2.2 Statistical analysis

Statistical analyses were performed using IBM SPSS version 21.0 (SPSS Inc, Chicago, IL). Descriptive statistics were calculated and unpaired *T* tests and Chi-squared tests were conducted when comparing continuous and categorical variables, respectively. Changes in time trend between 2000 and 2013 were examined by regression analysis. A *P*-value of .05 was considered significant.

## 3 Results

### 3.1 Number of articles

**Figure 2:**
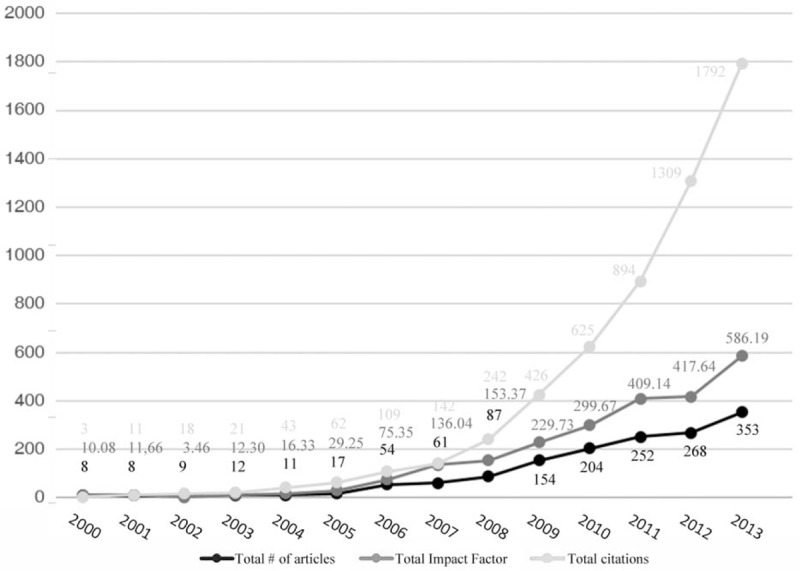
Trend for the total number of articles, total impact factor, and total number of citations over the study period.

A total of 1498 articles were identified and included in this study. The number of publications per year increased exponentially over the study period (*P* < .001, Fig. 2). And in absolute numbers, it grew by 44 times from year 2000 (8 articles) and year 2013 (353 articles). A more significant growth was observed with the total number of citations per year over the same period (*P* < .001). The total citations increased from year 2000 (3 citations) to year 2013 (1792 citations).

### 3.2 Impact factor

**Figure 3:**
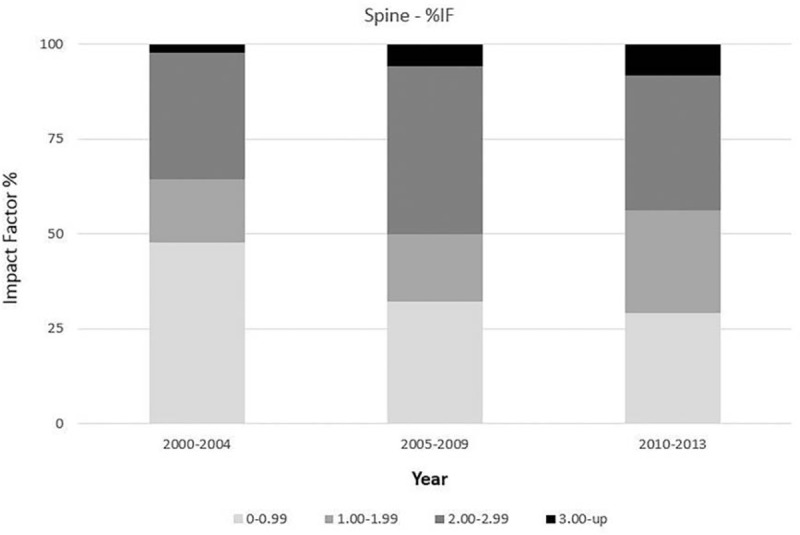
Comparison on the percentages of articles with different levels of impact factors over the study period.

The trend of annual IF changes paralleled that of the number of publications during the study period (Fig. 2). The average IF grew from 1.26 in 2000 to 1.66 in 2013. The average IF of articles published between 2000 and 2006 (mean = 1.25) was significantly lower than that of articles published between 2007 and 2013 (mean = 1.68, *P* = .042). Proportions of articles according to their IF (0–0.99, 1.00–1.99, 2.00–2.99, 3.00 and above) were compared (Fig. 3) and the difference between 2000 to 2004 (mean = 1.12), 2005 to 2009 (mean = 1.67), and 2010 to 2013 (mean = 1.59) was significant (*P* < .001). The top 8 journals with the highest number of publications by authors from mainland China were listed along with their associated IF (Table 1). Together they accounted for 62.3% of all published studies.

**Table 1:**
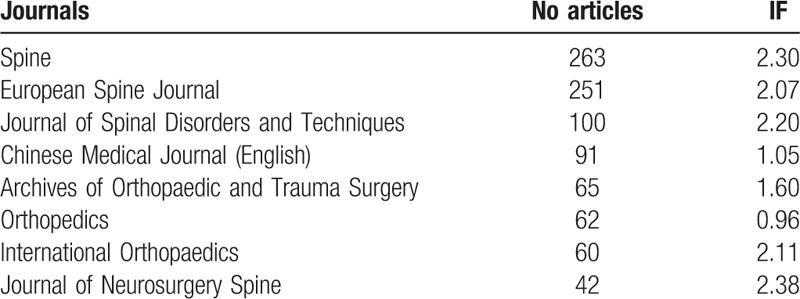
The 8 most popular journals and their associated impact factor (IF).

### 3.3 Geographical distribution

**Figure 4:**
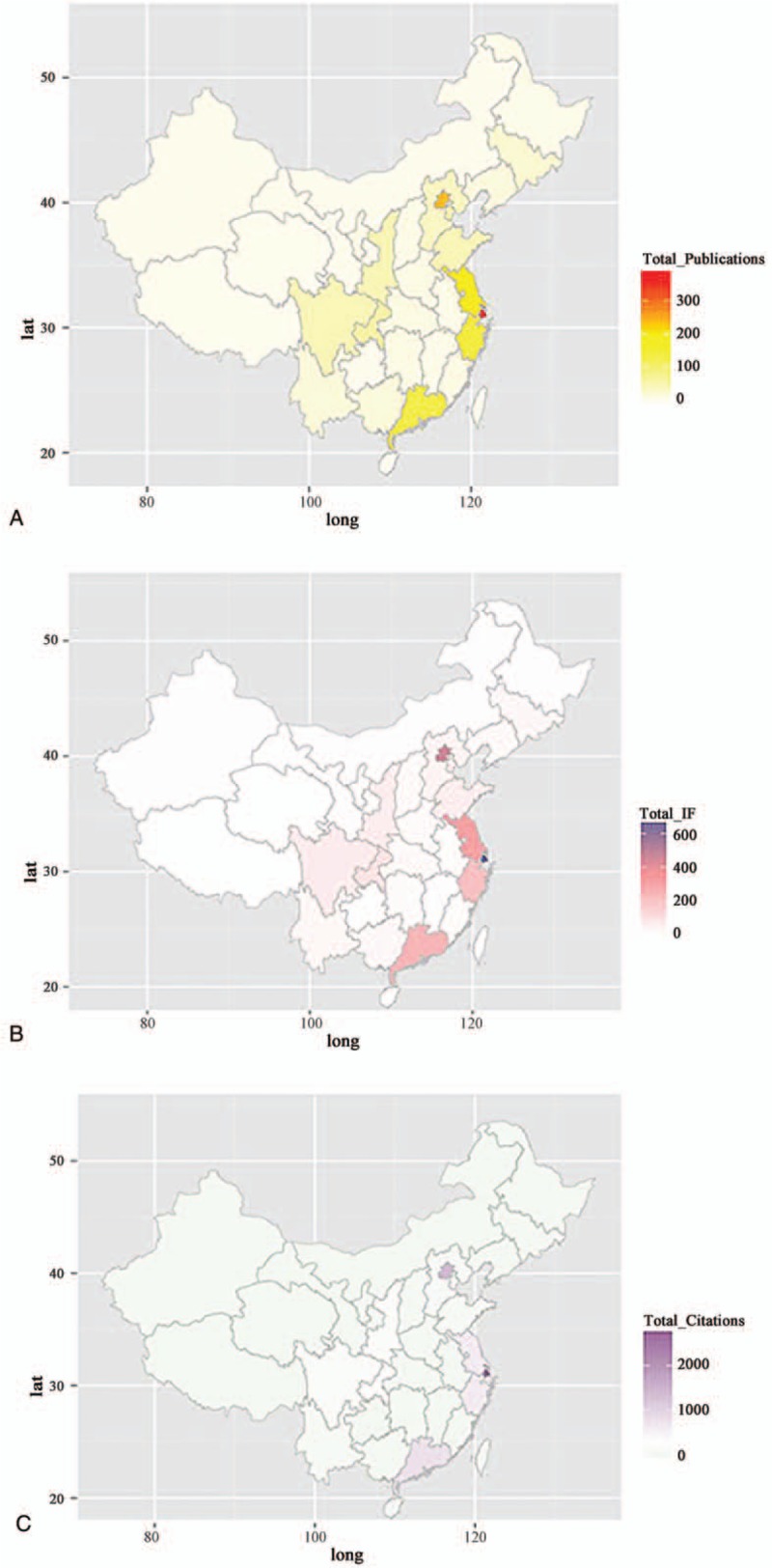
Geographical distribution of the total number of publications (A), total impact factor (B), and the total number of citations (C).

The total number of articles, total IF, and the total number of citations were calculated for each administrative division in mainland China to quantify their individual contribution (Fig. 4A–C). Shanghai and Beijing were top on the list by a large margin compared to all other regions in all 3 aspects (Table 2), indicating their unique status as hubs for clinical research. The second tier was the coastal provinces including Jiangsu, Zhejiang, and Guangdong. The third tier regions, such as Chongqing, Tianjin, Sichuan, Shaanxi, and Shandong, presented relatively better performance in their total number of articles (Fig. 4A) and annual IF (Fig. 4B) than in the total number of citations (Fig. 4C), possibly as a result of the increase in scientific publication in more recent years not yet being sufficiently accounted for by citation numbers. The fourth tier was composed of the least developed provinces in the northern and western parts of China.

**Table 2:**
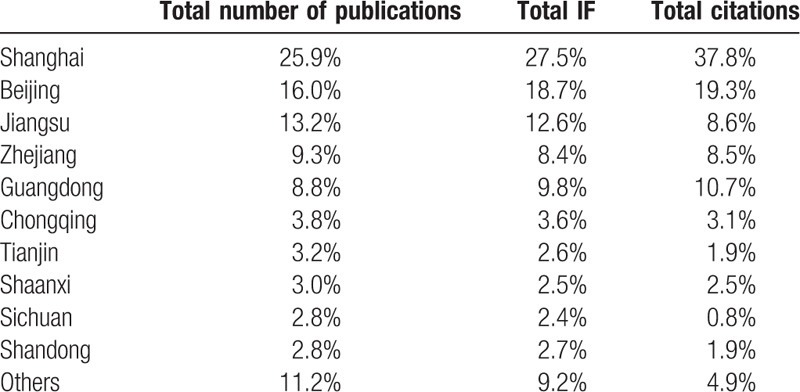
Annual number of publications, annual impact factors (IFs), and annual citations of different administrative regions.

### 3.4 Medical subject headings

**Figure 5:**
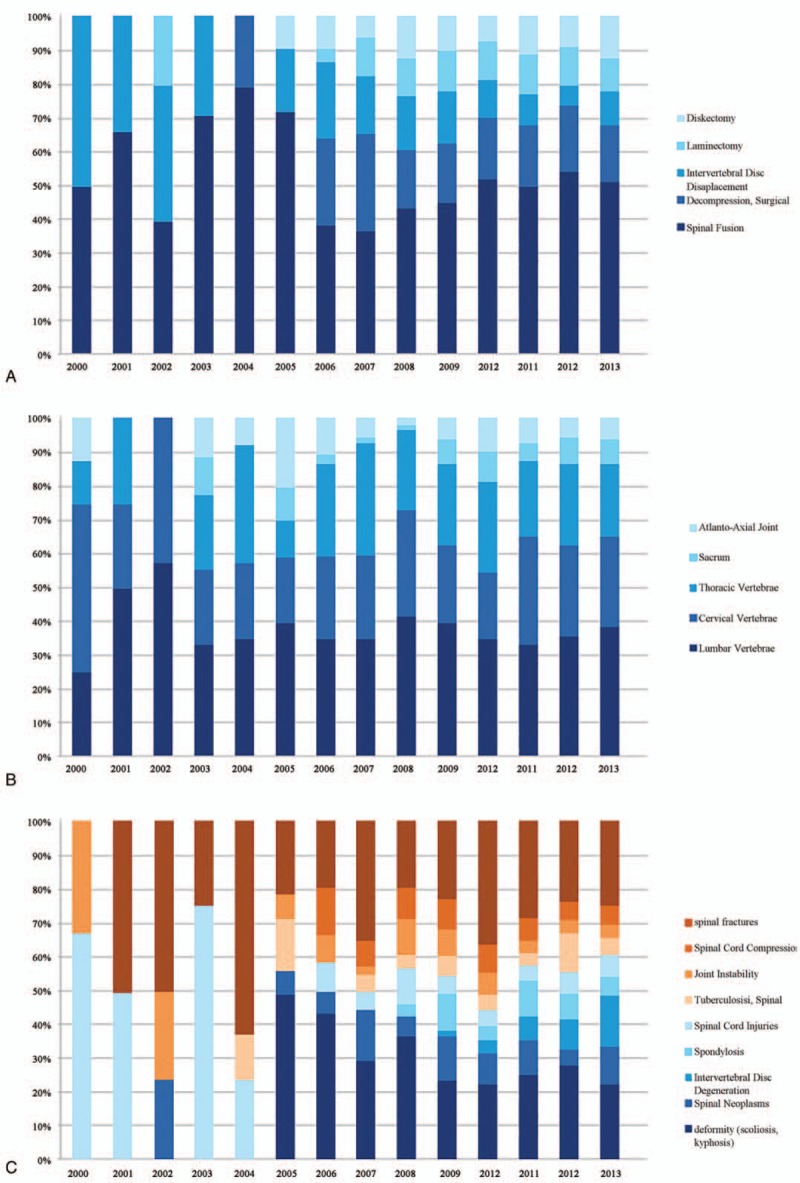
Comparison on the percentages of articles on various procedures (A), anatomical region (B), and disease conditions (C).

Medical subject headings (MeSHs) associated with each article were identified and ranked by descending frequency (top 20 shown in Table 3). Among the 50 most frequently used terms, the comparative frequencies of the identified specific procedures (“Discectomy,” “Laminectomy,” “Intervertebral disc replacement,” “Decompression, Surgical,” and “Spine fusion”), spinal regions (“Atlanto-axial joint,” “Cervical vertebrae,” “Thoracic vertebrae,” “Lumbar vertebrae,” and “Sacrum”), and the underlying pathologies (“Deformity (scoliosis, kyphosis),” “Spinal neoplasms,” “Intervertebral disc degeneration,” “Spondylosis,” “Spinal cord injuries,” “Tuberculosis, Spinal,” “Joint instability,” “Spinal cord compression,” “Spinal fractures”) are demonstrated in Figure 5. Specifically, “Spinal fusion” was the most frequently used MeSH and there was a decrease in research related to “Intervertebral disc displacement” over recent years (Fig. 5A). In terms of the spinal region under study, the lumbar, cervical, and thoracic spine accounted for 30% of all publications, respectively, and the atlanto-axial region and the sacrum combined were studied in 10% of published studies (Fig. 5B). Additionally, the spinal pathologies under research became more diverse during the study period, with no more than 4 being represented before 2004 and all 9 different pathologies studied since 2009 (Fig. 5C). Furthermore, while “Spinal cord injury” was a topic of primary research interest before 2004, studies on “Deformity (scoliosis, kyphosis)” and “Intervertebral disc degeneration” had become more popular subjects of study since then (Fig. 5C).

**Table 3:**
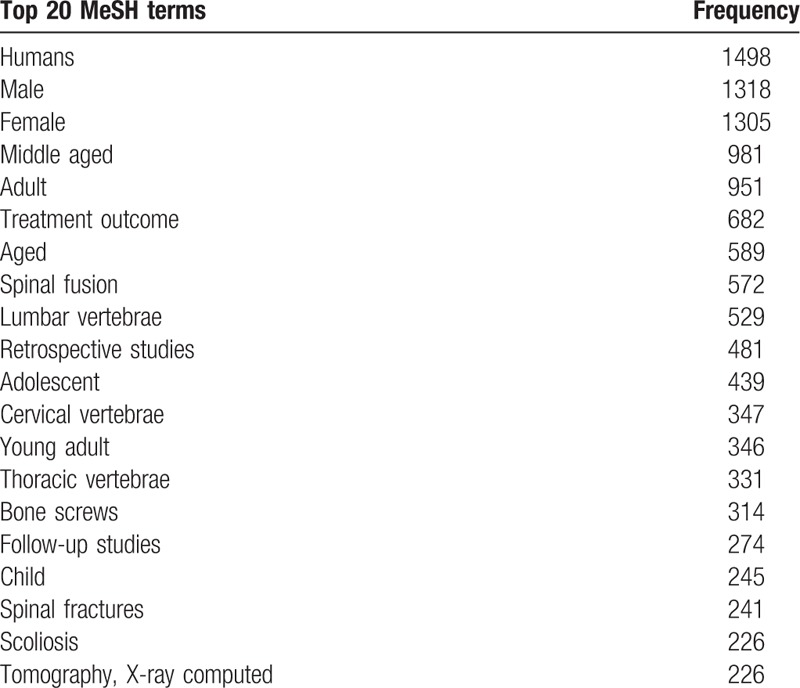
The 20 most frequently used medical subject headings (MeSHs).

## 4 Discussion

Bibliometric study is a significant method to access the development trend and research hotspot of an academic field or discipline specialty, which is very helpful to grasp the research trends in the field. Based on the comprehensive analysis of publications’ bibliographic data, to a certain extent, the increase of the scientific literature number reflects the characteristics and laws of academic research of a subject or special topic in a certain period of time. Our present study represented the first comprehensive evaluation on the quantity and quality of clinical research regarding spine surgery from mainland China, which reflected the contribution of mainland China to the global orthopedic spine surgery research, and helped to analyzed the existed problems, provides a reference for future-related research on this topic.

Total of 1498 articles were identified, through the bibliometric analysis of orthopedic spine surgery research from mainland China, the annual number of publications, citations, and IFs all increased exponentially. The average IF was significantly higher in 2007 to 2013 than 2000 to 2006. Most publications were from Shanghai and Beijing and the 5 most productive administrative regions. “Spinal fusion” was the most frequently used MeSH and there was a decrease in research related to “Intervertebral disc displacement” over recent years. Also, the spinal pathologies under research became more diverse during the study period.

With increasing support from devoted resources and funding, output of clinical research in this field has increased dramatically,^[21]^ similar to what was found in previous reports in critical care medicine,^[22]^ plastic surgery,^[23]^ anesthesiology,^[24]^ infectious disease, respiratory,^[25]^ and other clinical entities. Specifically, although previous studies based on analyses of a shortlist of general orthopedic or spine journals have demonstrated significant progress from mainland China during the past decade,^[26–28]^ to our knowledge, this is the first study to provide an overview of spine surgery-related clinical research output by Chinese physician-scientists as published in all biomedical journals. In fact, the fourth most common venue of publication (with 91 articles) identified herein was a general medical journal that was not reported in previous studies. In other words, if only orthopedic and spine journals were investigated, at least 6% of studies would have been missed.

In addition, quality of the identified clinical research studies has also improved significantly as measured by their average IF.^[29,30]^ The exponential growth rate associated with the annual IF was even higher than that of the annual total number of articles since 2008. Furthermore, some of the researches can be influential even worldwide.^[15,31]^ The trend for more high-impact research was also demonstrated by the decreasing proportion of low-impact (IF < 1.00) studies in recent years. The IF of the 3 most popular journals (all with more than 100 studies published from mainland China) were all above 2.00, further indicating the increasing proportion of high-impact research from mainland China authors.

Analysis on the geographical distribution of scientific output within mainland China found Shanghai and Beijing as 2 hubs for spine surgery-related clinical research in all aspects examined in this bibliometric study and the 5 most productive administrative divisions (Shanghai, Beijing, Jiangsu, Zhejiang, and Guangdong) accounted for about 70% of the total number of publications, 75% of the total IF, and 85% of all citations. This was in agreement with previous findings both in the field of other subspecialties.^[17,18,32–34]^ While this had to do with historical and cultural reasons, the impact of faster economic and social development during the past decades^[35]^ and subsequent concentration of supporting resources and availability of research funding in these regions were evident.^[36]^ These numbers, when contrasted to the percentage of the population represented by these regions (20.9%), illustrated the disparity in allocation of health care resources and the resulting conflicts of accessibility, affordability, and quality that were rampant in the health care system in China.^[37]^ While it was arguable that scientific productivity did not necessarily always correlate with patient volume in a certain institute, given the current circumstances in mainland China where spine procedures were rarely performed in a private practice setting, and academic performance remained as a major index for professional evaluation in public hospitals,^[38]^ it was quite likely that the uneven distribution of clinical research publications reflected the actual disparity in the availability of health care resources in different regions.^[39,40]^

MeSHs were used by the National Library of Medicine as a manually assigned controlled vocabulary to describe the central concepts that were discussed in MEDLINE articles. While originally designed to facilitate information retrieval and text-mining, it could also be used in analysis of characteristics of study subjects. Although “spinal fusion” remained the most common concept under investigation throughout the study period, clinical scientists from mainland China had published on a more heterogeneous group of spinal pathologies in recent years.

Previous studies suggested improved English proficiency was correlated with higher scientific productivity.^[41,42]^ Although reasonable, this impact was hard to quantify and was therefore not evaluated in this study. None of the articles included was published by private institutions and there was no private source of funding reported. While it could have added to the overall interest of this study, it was not feasible to demonstrate the distribution of randomized controlled trials, prospective studies, retrospective comparative studies, case series, and review articles, due to insufficiency of relevant information. It was similarly not feasible to compare the percentage of government-funded versus unfunded research. Furthermore, even though there was the issue of accessibility (with people frequently having to travel hundreds of miles for better medical care), the concentration of spine service in a handful of centers could actually facilitate subject recruitment in clinical trials. And lastly, it was worthy to point out that IFs and citation numbers were only imperfect indices for the real impact and significance of a research project, but they were nonetheless widely used due to lack of better alternatives.

## 5 Conclusion

To conclude, this first comprehensive evaluation of the contribution of spine-surgery-related clinical research output from mainland China demonstrated exponential growth in the total number of annual publications, annual total IFs, and the annual total number of citations between 2000 and 2013. Clinical research output regarding spine surgery was highly concentrated in a handful of administrative regions in mainland China. These data are helpful to domestic scientific researchers in research planning and decision making of this field. The present study can also help researchers to find research hot spots and gaps on this topic, and improve the efficiency of research output.

## Author contributions

**Conceptualization:** Gao Si, Xiao Liu, Nanfang Xu, Miao Yu, Xiaoguang Liu.

**Data curation:** Gao Si, Xiao Liu, Nanfang Xu, Miao Yu.

**Formal analysis:** Gao Si, Xiao Liu.

**Investigation:** Gao Si.

**Visualization:** Nanfang Xu.

**Writing – original draft:** Gao Si.

**Writing – review & editing:** Gao Si.
